# Supplementary material for: Composite Meal-Related Behaviors and Health Indicators: Insight from Large-Scale Nationwide Data on Korean Adults
Source: Nutrients. 2025 Sep 17;17(18):2982. doi: 10.3390/nu17182982 (PMC12472365; doi:10.3390/nu17182982)
Supplement: Supplementary file 1 [file nutrients-17-02982-s001.zip › nutrients-3846544-supplementary.pdf]

Table S1. Multinomial logistic regression analysis of factors associated with meal-related behavior status among young adults.

Variable	Dietary status	OR	95% CI Lower	95% CI Upper
Sex, male	Moderate	2.02	1.69	2.41
	Low	2.59	2.14	3.12
Body mass index	Moderate	1.29	0.97	1.49
	Low	1.08	0.96	1.29
Living alone, yes	Moderate	1.82	1.69	2.39
	Low	2.46	2.01	3.57
Basic Livelihood Security, yes	Moderate	0.94	0.73	1.22
	Low	1.02	0.78	1.33
Hypertension, yes	Moderate	1.02	0.64	1.61
	Low	0.94	0.59	1.51
Dyslipidemia, yes	Moderate	1.00	0.66	1.50
	Low	0.82	0.54	1.25
Diabetes mellitus, yes	Moderate	0.65	0.31	1.35
	Low	1.40	0.63	2.10
Sedentary time	Moderate	1.06	1.04	1.08
	Low	1.20	1.08	1.11
Fasting glucose	Moderate	0.99	0.99	1.00
	Low	1.71	1.53	1.91
Total cholesterol	Moderate	1.00	0.99	1.00
	Low	1.00	0.99	1.00
Triglycerides	Moderate	1.00	0.99	1.00
	Low	1.00	0.99	1.00
Total energy intake, kcal	Moderate	1.00	1.00	1.00
	Low	1.43	1.28	1.61
Carbohydrate intake	Moderate	1.09	0.99	1.12
	Low	1.01	0.97	1.09
Protein intake	Moderate	0.99	0.99	1.00
	Low	1.99	1.69	2.13
Fat intake	Moderate	1.00	0.99	1.00
	Low	1.55	1.39	1.93
Cholesterol intake	Moderate	1.09	0.97	1.18
	Low	1.00	0.99	1.00
Saturated fatty acid intake	Moderate	1.00	0.99	1.01
	Low	1.00	0.99	1.01
Dietary fiber intake	Moderate	0.76	0.69	0.88
	Low	0.65	0.55	0.71

Odds ratios (OR) and 95% confidence intervals (CI) are shown for each variable, with the reference group being the High adherence group.

Table S2. Multinomial logistic regression analysis of factors associated with meal-related behavior status among middle-aged adults.

Variable	Dietary status	OR	95% CI Lower	95% CI Upper
Sex, male	Moderate	2.29	2.07	2.53
	Low	3.08	2.71	3.50
Body mass index	Moderate	0.99	0.98	1.00
	Low	1.01	0.99	1.02
Living alone, yes	Moderate	2.89	1.99	3.88
	Low	3.12	2.57	4.01
Basic Livelihood Security, yes	Moderate	1.07	0.92	1.25
	Low	1.06	0.87	1.28
Hypertension, yes	Moderate	1.18	1.08	1.29
	Low	1.81	1.65	2.59
Dyslipidemia, yes	Moderate	1.19	1.09	1.30
	Low	2.13	1.78	2.98
Diabetes mellitus, yes	Moderate	1.03	0.88	1.19
	Low	1.62	1.41	1.88
Sedentary time	Moderate	1.04	1.03	1.05
	Low	1.57	1.43	1.78
Fasting glucose	Moderate	1.00	0.99	1.00
	Low	1.00	0.99	1.00
Total cholesterol	Moderate	1.00	1.00	1.00
	Low	2.01	1.78	3.11
Triglycerides	Moderate	1.00	1.00	1.00
	Low	2.25	1.82	3.41
Total energy intake, kcal	Moderate	1.02	1.00	1.10
	Low	1.12	1.09	1.29
Carbohydrate intake	Moderate	0.99	0.99	0.99
	Low	0.99	0.99	0.99
Protein intake	Moderate	1.00	0.99	1.00
	Low	0.99	0.99	1.00
Fat intake	Moderate	0.99	0.99	1.00
	Low	2.31	1.95	2.91
Cholesterol intake	Moderate	1.00	1.00	1.00
	Low	2.29	1.82	3.08
Saturated fatty acid intake	Moderate	1.00	0.99	1.01
	Low	1.86	1.74	2.39
Dietary fiber intake	Moderate	0.89	0.78	0.99
	Low	0.77	0.69	0.81

Odds ratios (OR) and 95% confidence intervals (CI) are shown for each variable, with the reference group being the High adherence group.

Table S3. Multinomial logistic regression analysis of factors associated with meal-related behavior status among older adults.

Variable	Dietary status	OR	95% CI Lower	95% CI Upper
Sex, male	Moderate	0.97	0.85	1.09
	Low	1.56	1.23	1.98
Body mass index	Moderate	1.02	0.97	1.04
	Low	1.01	0.98	1.04
Living alone, yes	Moderate	3.11	2.81	3.74
	Low	4.35	4.09	4.76
Basic Livelihood Security, yes	Moderate	1.49	1.25	1.77
	Low	2.11	1.63	2.74
Hypertension, yes	Moderate	1.01	0.91	1.12
	Low	1.11	0.91	1.35
Dyslipidemia, yes	Moderate	1.07	0.96	1.19
	Low	0.82	0.67	1.01
Diabetes mellitus, yes	Moderate	0.91	0.79	1.05
	Low	1.07	0.81	1.42
Sedentary time	Moderate	1.02	1.01	1.04
	Low	1.54	1.41	1.66
Fasting glucose	Moderate	1.00	0.99	1.00
	Low	1.00	0.99	1.00
Total cholesterol	Moderate	1.00	0.99	1.00
	Low	1.71	1.64	1.92
Triglycerides	Moderate	1.00	0.99	1.00
	Low	1.00	0.99	1.00
Total energy intake, kcal	Moderate	1.00	1.00	1.00
	Low	0.79	0.61	0.86
Carbohydrate intake	Moderate	0.99	0.99	1.00
	Low	0.84	0.79	0.91
Protein intake	Moderate	0.99	0.99	1.00
	Low	0.86	0.79	0.96
Fat intake	Moderate	0.99	0.99	1.00
	Low	1.00	0.98	1.01
Cholesterol intake	Moderate	1.00	0.99	1.00
	Low	1.00	1.00	1.00
Saturated fatty acid intake	Moderate	1.00	0.98	1.02
	Low	1.02	0.99	1.06
Dietary fiber intake	Moderate	0.99	0.99	1.09
	Low	0.71	0.67	0.89

Odds ratios (OR) and 95% confidence intervals (CI) are shown for each variable, with the reference group being the High adherence group.
